# Supplementary material for: Form factor determination of biological molecules with X-ray free electron laser small-angle scattering (XFEL-SAS)
Source: Commun Biol. 2023 Oct 18;6:1057. doi: 10.1038/s42003-023-05416-7 (PMC10585004; doi:10.1038/s42003-023-05416-7)
Supplement: Supplementary file 2 — Supplementary figures [file 42003_2023_5416_MOESM2_ESM.pdf]

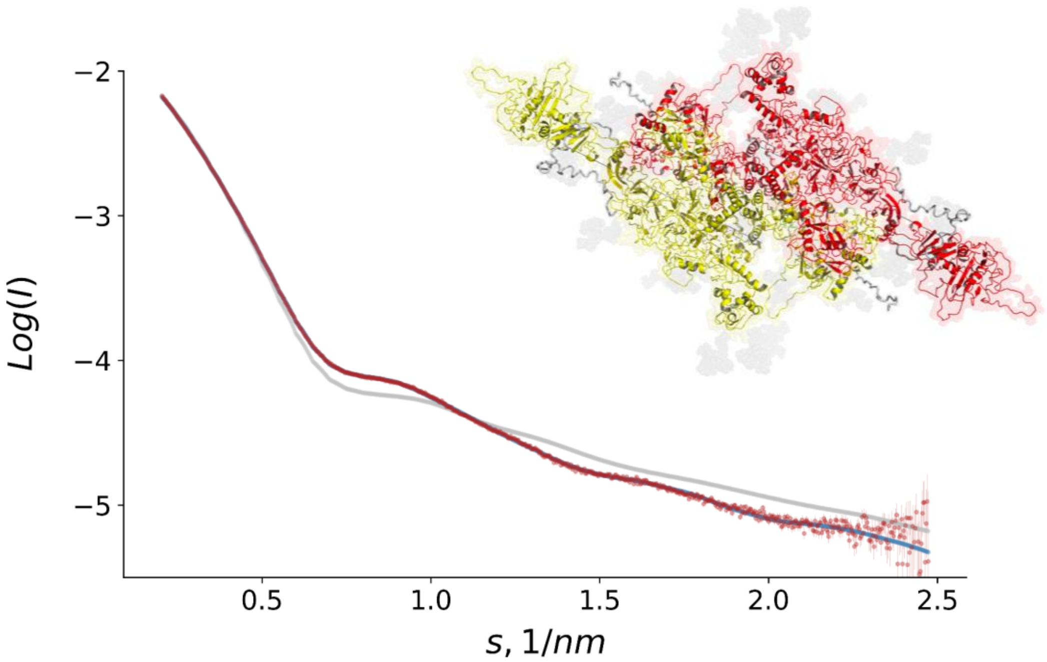

Figure S1: Application of advanced modelling procedures to XFEL-SAS data of Bovine Thyroglobulin. In grey the scattering curve computed from the cryoEM model (PDB ID: 6SCJ) and where residues missing at the N/C terminal and in two linkers have been added. In blue, with the glycans added and refined against the experimental SAXS data (red) using the program CORAL. Insert: Coral model, each dimer is of different colour red/yellow, glycosylated parts are shown as semi-transparent grey beads.

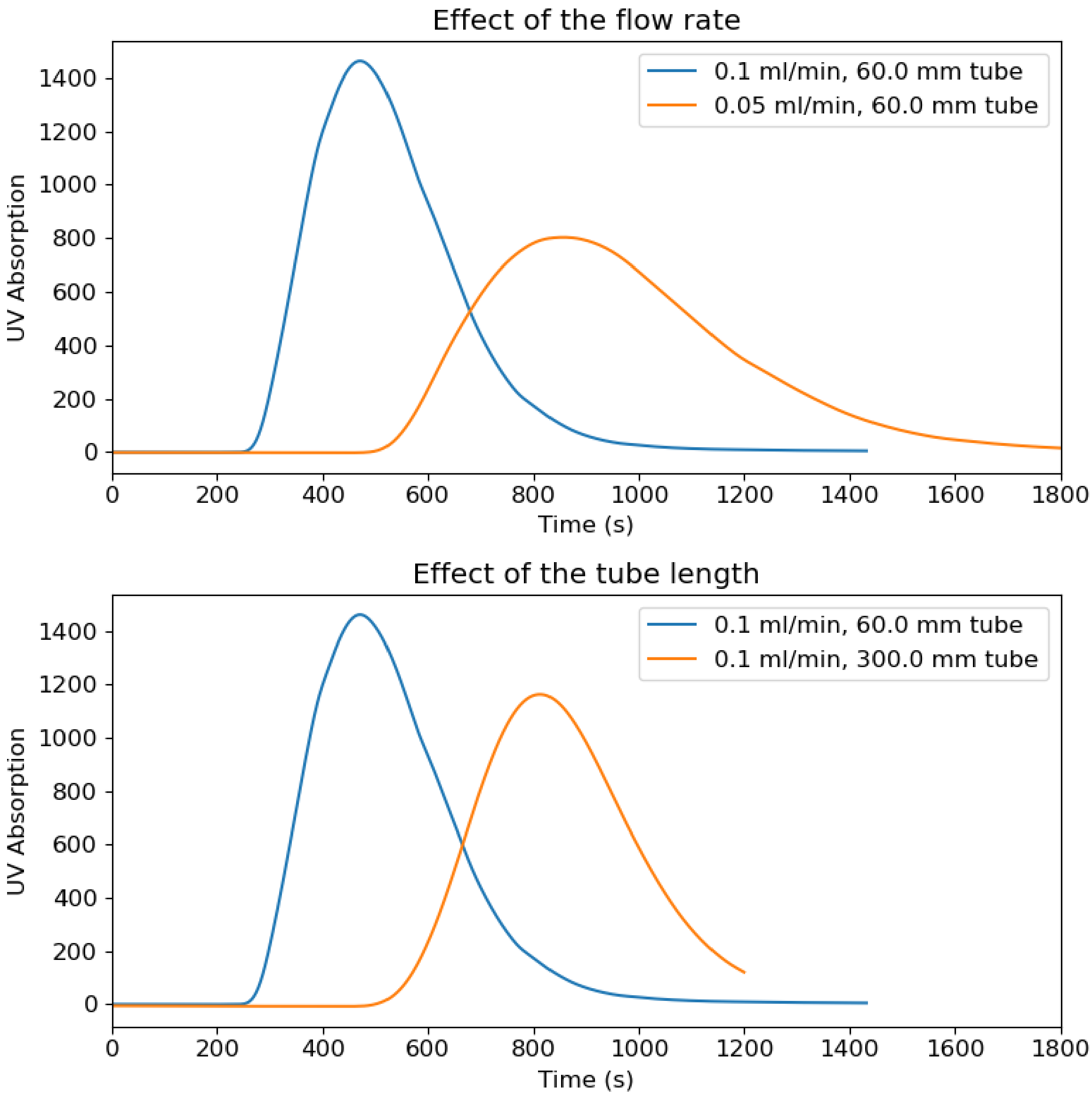

Figure S2: Dilution of the protein samples along the delivery line in function of the flow rate and tube length. 20µl of sample were injected into the flow at different velocity (0.1 and 0.05 ml/min) through tube of different length (60 and 300 mm). UV absorption traces, scaling with the protein concentration were collected after the tube. The longer time the proteins spend in the transfer line (due to lower flow velocity or longer tube), the more it gets diluted. While the sample is diluted, the protein concentration decreases but remains within the same order of magnitude.

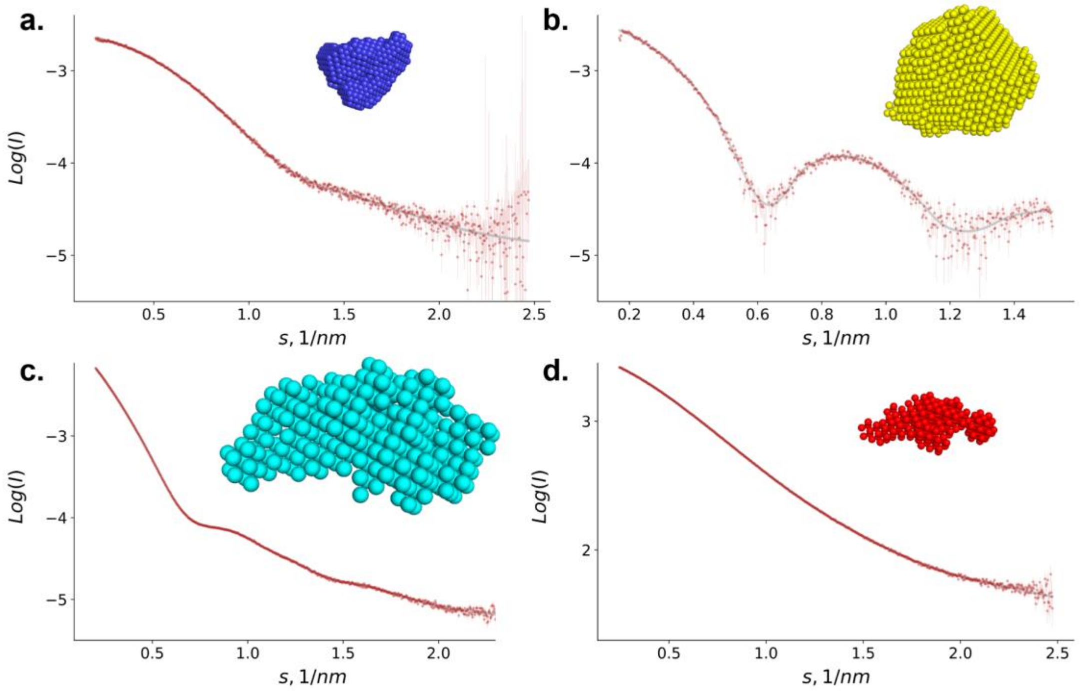

Figure S3: *Ab-initio* model reconstruction from XFEL data and corresponding fit for BSA (**a.**), Apoferritin (**b.**), Thyroglobulin (**c.**) and spike RBD (**d.**). Models were built from the autosampler data for BSA and apoferritin, and reservoir data for thyroglobulin and spike RBD.

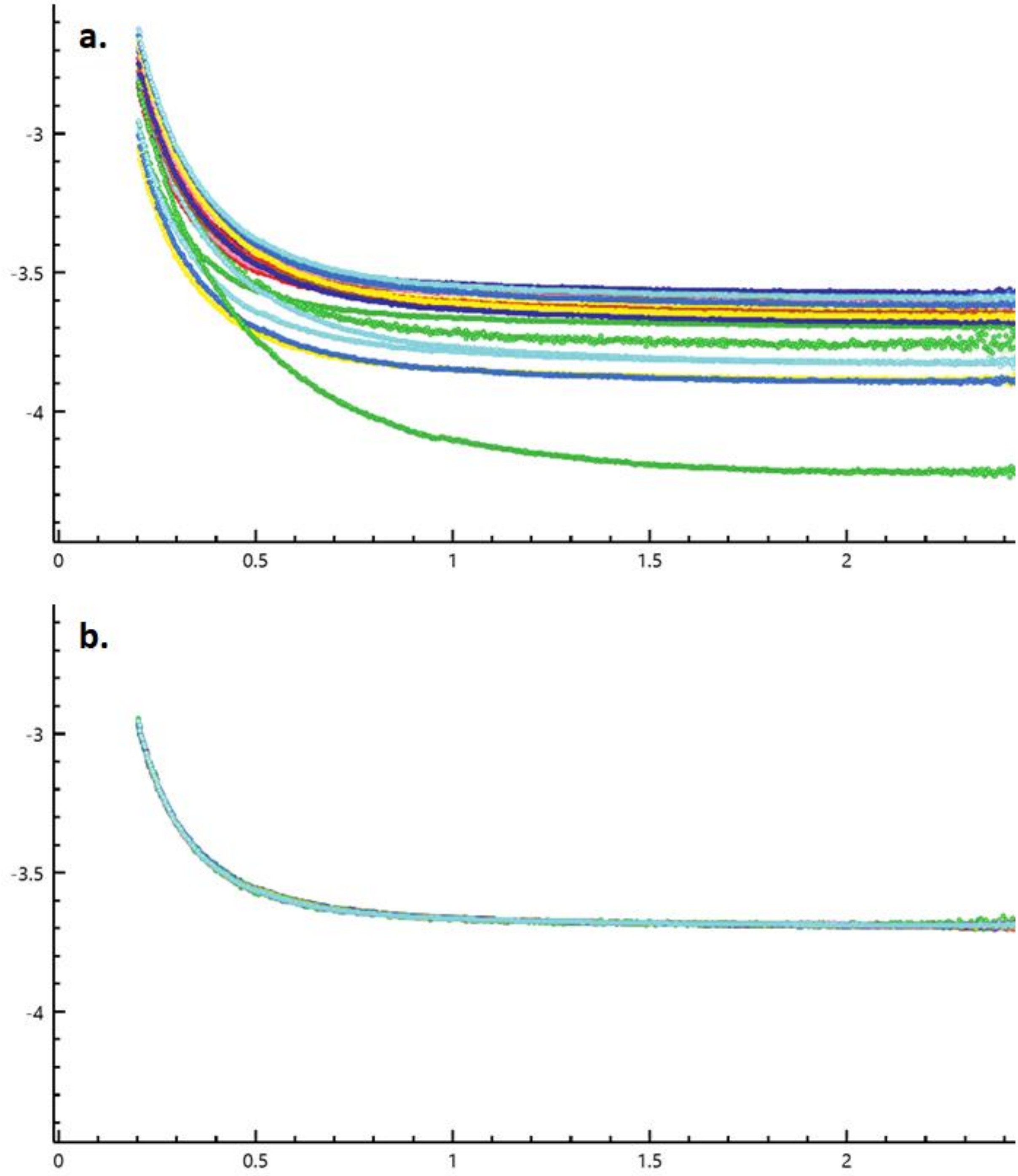

Figure S4: Curves collected on PBS buffer using reservoir delivery during the second day of measurement. (a) Individual curves after radial averaging show noticeable variation, likely attributed to differences in alignment, jet parameters, and total exposure time. (b) All curves were adjusted using a scaling factor to account for changes in beam intensity and a constant offset to compensate for variations in sample thickness (jet diameters). Remarkably, the adjusted curves exhibit significant overlap, suggesting that despite differences in beam intensity and jet diameter, the acquired data remain consistent and reliable. These variations in the curves are mainly caused by fluctuations in the beam intensity (accounted for, mostly, by the scaling factor) and liquid jet diameter (resulting in a constant offset) during data collection.

The use of an autosampler allows for collection of sample and buffer within the same run, minimizing the effect of changes in beam intensity and jet diameter, and eliminating the need for curve adjustment. This advantage of the autosampler significantly contributes to the robustness and reliability of our measurements, providing more accurate insights into the structural properties of the biological samples.

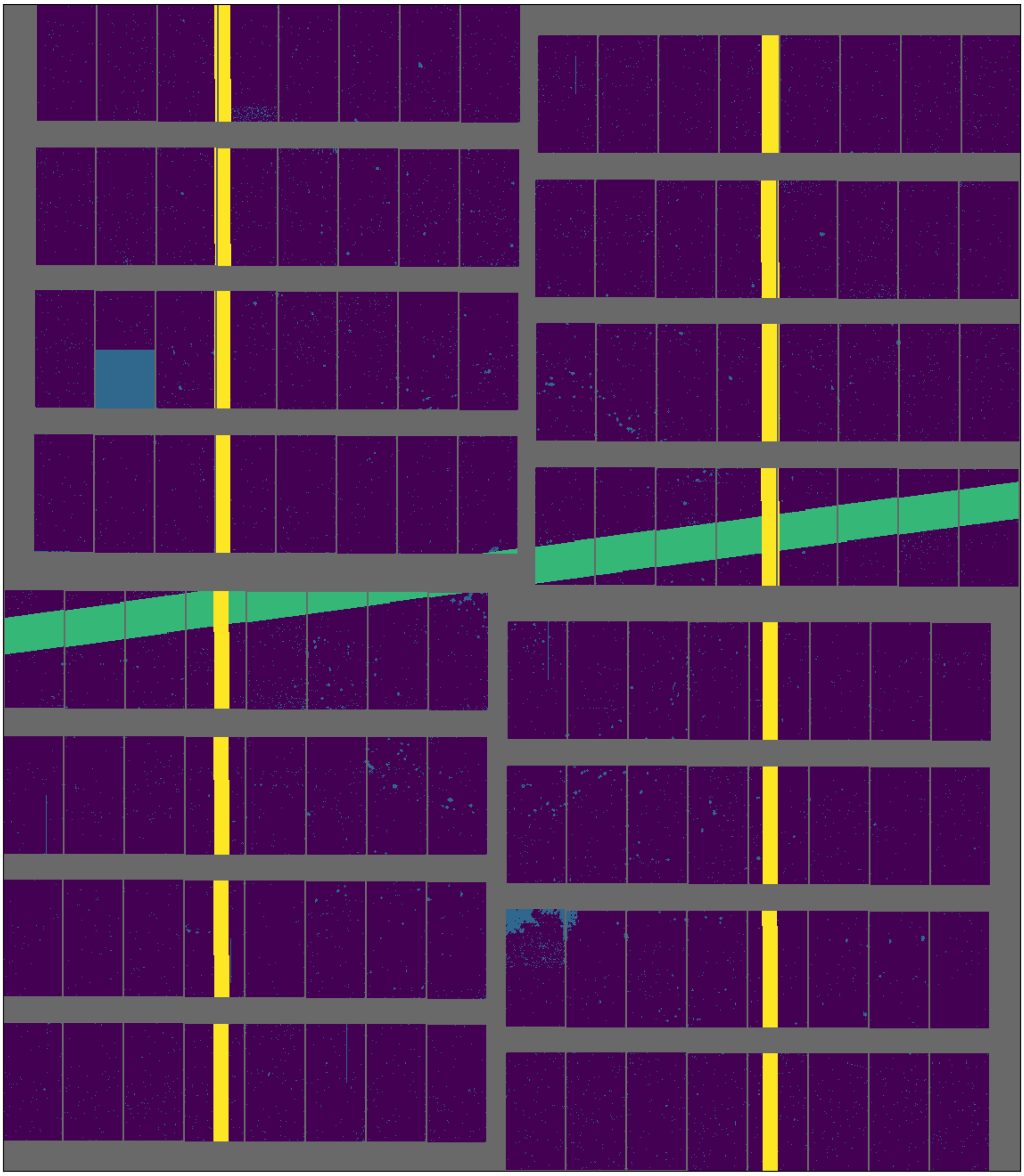

Figure S5: Image illustrating the position of the beam (red cross), the detector modules and the masked pixels. Dark blue: Good pixels, Turquoise: Bad pixels (different for each AGIPD cell, here shown for cell 1), Light green: Jet streak, Yellow: Shadowed region (for baseline correction).

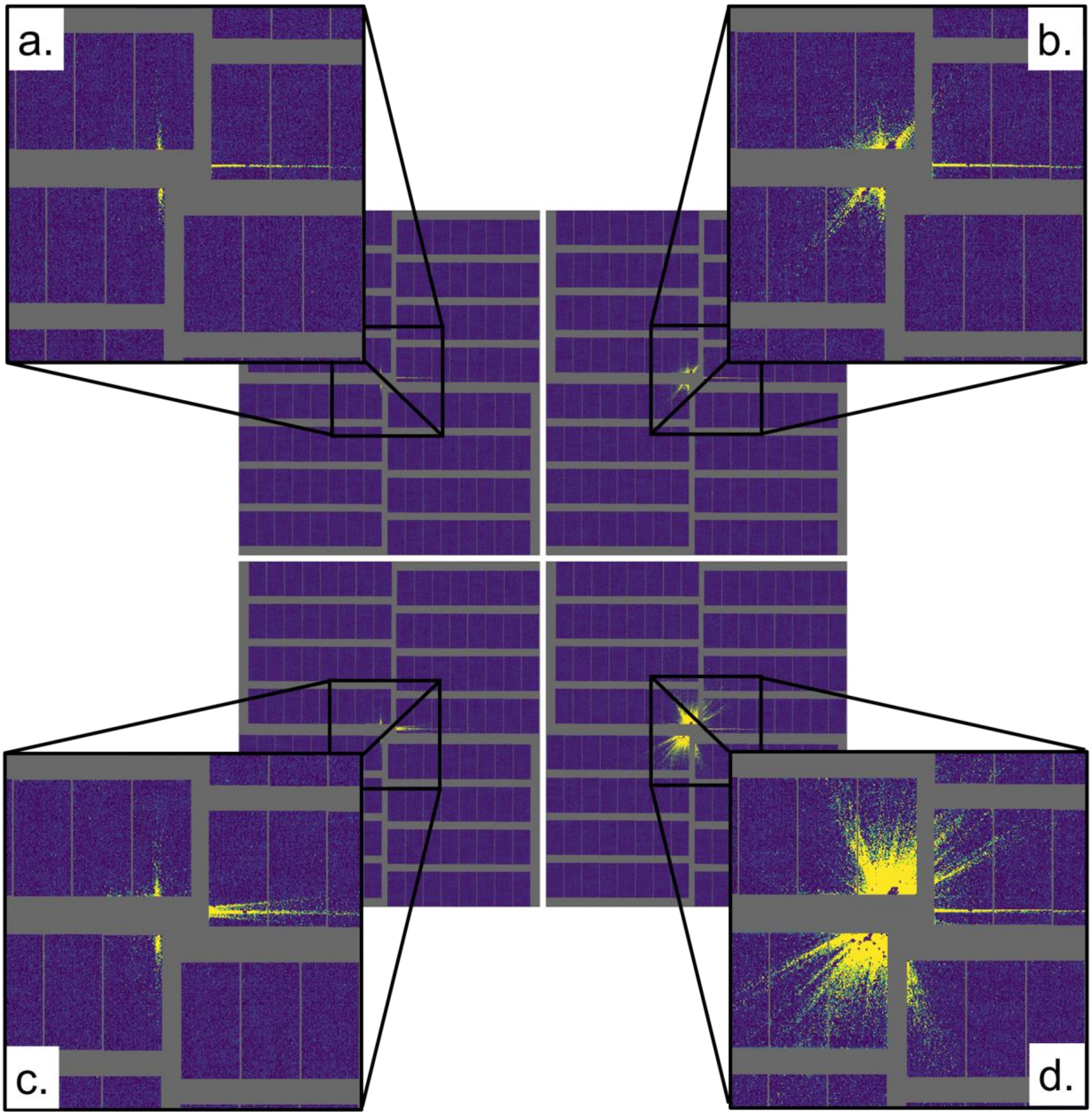

Figure S6: Example of frames accepted (a.) and rejected (b., c. and d.) by the filtering algorithm. While some residual parasitic scattering originating from the slits and possibly the jet edge can still be observed in image A, it is confined to the vicinity of the direct beam and can be readily excluded through masking. In contrast, images B, C, and D exhibit significantly higher and more dispersed levels of parasitic scattering, suggesting jet breakup or misalignment. This scattered signal cannot be masked and, consequently, those frames are filtered out from the analysis.
